# An unusual case of low-grade tubulopapillary adenocarcinoma of the sinonasal tract

**DOI:** 10.1186/1477-7819-6-54

**Published:** 2008-05-20

**Authors:** Ashish Bansal, Keloth E Pradeep, Krishna P Gumparthy

**Affiliations:** 1 Department of Histopathology, Wirral Hospitals NHS Trust, Upton, Wirral, CH49 5PE, UK; 2 Department of Histopathology, Wrexham Maelor Hospital, Wrexham, UK

## Abstract

**Background:**

Low-grade papillary adenocarcinomas of the sinonasal tract are rare neoplasms. Over recent years, little doubt remains that this tumour represents a separate entity based on morphology, ultrastructural features and behaviour. We outline a case of this rare entity displaying a not hitherto described immunophenotype.

**Case presentation:**

A 32 year old man presented recurrent epistaxis was evaluated with endoscopy which revealed a well circumscribed pedunculated mass lesion in left nares. The mass was arising from the nasal septum which was excised along with the mass. The biopsy revealed low-grade, non-intestinal type sinonasal tubulopapillary adenocarcinoma.

**Conclusion:**

TTF-1 immunoreactivity in absence of thyroid or pulmonary primary in the present case remains an enigma. However, this raises the possibility of the utility of this antibody to predict a better clinical outcome in the subset of low grade non-intestinal sinonasal adenocarcinoma. More cases of similar morphological appearance may need to be examined for TTF-1 immunoreactivity and clinically followed up to establish this theory.

## Background

Sinonasal adenocarcinomas are rare tumours accounting for 0.4% [1] of all human neoplasms, of which adenocarcinoma accounts for 13% [2]. We outline a case of this rare entity displaying an unusual immunophenotype.

## Case presentation

A 32 year old man who had recurrent episodes of epistaxis was seen in the ENT outpatient clinic. Flexible endoscopy revealed deviation of the nasal septum to the left. Arising from the posterior end of the left nasal septum was a pedunculated well-circumscribed lesion. Magnetic resonance imaging revealed no other abnormalities. At operation, a lobulated solid mass was seen. The mucosa anterior to the mass had become detached. The underlying bone was removed but did not look involved. Postoperative recovery was uneventful and he was discharged the next day. The lesion was suspected to be a haemangioma. Previous episodes of epistaxis were treated with silver nitrate cautery. The patient has no significant past medical history. He is a non-smoker, was not on any regular medication and had no relevant occupational history. Subsequently, the patient had two further operations. Firstly, removal of the posterior aspect of the nasal septum was performed four months after removal of this mass. Secondly, a biopsy of the nostril was undertaken. The former revealed mucosal fragments incorporating seromucinous glands with intervening chronic inflammation of the stroma but no evidence of residual adenocarcinoma. The latter showed inflammatory granulation tissue around suture granulomata from previous surgery. Since initial presentation over two years ago, the patient remains free of recurrence or metastatic disease and does not have any lesions in his lungs or thyroid gland.

**Figure 1:**
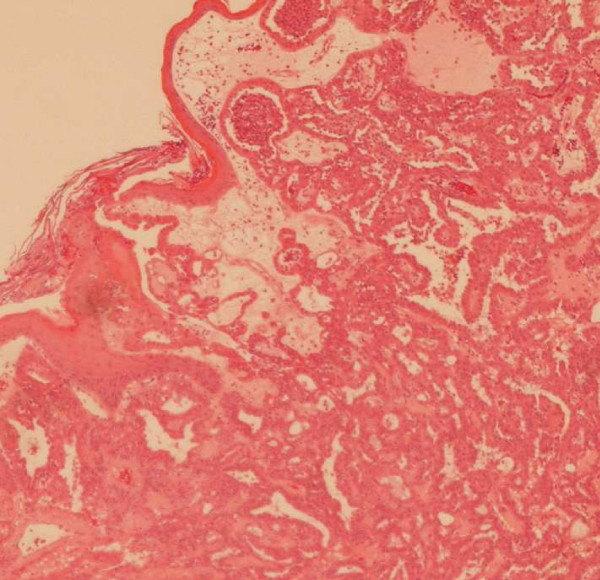
Low Power photomicrograph (×40) of this entity: low-grade non-intestinal tubulopapillary adenocarcinoma of the sinonasal tract with overlying surface squamous epithelium.

**Figure 2:**
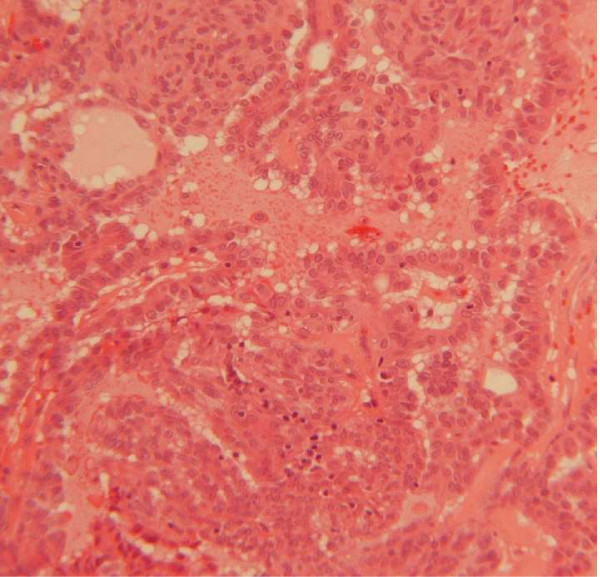
High power photomicrograph (×250): complex tubules and papillae lined by mild/moderately dysplastic pale columnar cells.

Macroscopically, two yellow-white polypoid fragments of tissue, measuring 10 and 4 mm in maximum dimension were received. Histologically, these fragments were partly covered by focally ulcerated squamous epithelium. The underlying stroma was infiltrated by a neoplasm with a complex papillary and tubular configuration, lined by moderately dysplastic pale columnar epithelium with intervening spindle shaped cells(Figure 1 and 2).

**Figure 3:**
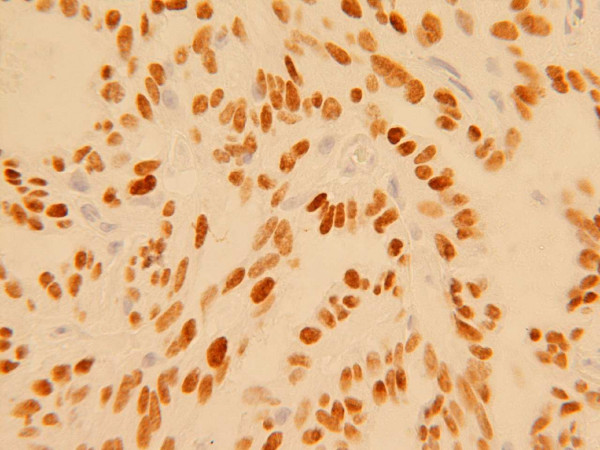
Immunohistochemical nuclear positivity for thyroid transcription factor 1 (TTF-1).

Immunohistochemical labelling revealed diffuse positivity with antibodies to EMA, CAM 5.2, CK 7, CK 19 and TTF-1 (Figure 3). The cells were negative with CK 20, CEA, S-100 protein, thyroglobulin, SMA and p63. The appearances were consistent with a low-grade, non-intestinal type sinonasal tubulopapillary adenocarcinoma.

## Discussion

As described recently [3], low-grade tubulopapillary adenocarcinoma represents a distinctive sinonasal adenocarcinoma. Historically, one of the earliest classifications was based on whether the tumour arose from the surface mucosal epithelium or from submucosal seromucinous glands [4]. However, this separation was flawed in that the latter are direct invaginations of the former. Subsequently, some pathologists began to classify these tumours solely as high-grade or low-grade adenocarcinomas based on their histological appearance [5]. In view of the histological resemblance of sinonasal adenocarcinomas to intestinal and submucosal seromucinous glands, classifications [6] have tended to categorise such tumours into intestinal and non-intestinal types. The current WHO classification [7] of these tumours considers two categories: intestinal and non-intestinal types of high and low grade sub-types. In addition, sinonasal tumours of the salivary gland type are identified too. The high grade types in both groups of adenocarcinomas and the overall category of intestinal type are described to have a worse prognosis.

The importance of recognition and separation of this neoplasm from other types of sinonasal adenocarcinoma is critical as it virtually never metastasizes and has an excellent prognosis. Unlike this case, Franchi *et al*. [8], have recently described two cases positive for basal cell markers, demonstrating that at least a subset of these tumours are most likely salivary-type in origin. With the possible exception of a low proliferation index, immunohistochemical markers have so far proved unhelpful. Immunohistochemistry for intestinal type adenocarcinoma is known to reveal positivity for pancytokeratin, EMA, B72.3, BerEP4, Leu M1, CK20, CDX2 and variable CK7 immunoreactivity. In this case, the tumour showed diffuse positivity with antibodies to EMA, CAM 5.2, CK7, CK19 and TTF-1 and no expression (negative) with CK 20, CEA, S-100 protein, thyroglobulin, SMA and p63.

## Conclusion

There is no published data on the role of TTF-1 in adult primary nasal adenocarcinomas. To date, we are unaware of any occult thyroid or pulmonary tumours in our patient to explain the TTF-1 immunoreactivity. The significance of this unexpected immunohistochemical labelling remains an enigma. However, this unusual TTF-1 positivity raises the possibility of the utility of this antibody to predict a better clinical outcome in the subset of low grade non-intestinal sinonasal adenocarcinoma. More cases of similar morphological appearance may need to be examined for TTF-1 immunoreactivity and clinically followed up to establish this theory.

## Competing interests

The authors declare that they have no competing interests.

## Authors' contributions

AB conducted a literature search, took the photomicrographs and drafted the manuscript; KEP edited the manuscript; KPG is the consultant who reported the biopsies and proofread the final manuscript. All authors read and approved the final manuscript.
